# Race-Level Reporting of Incidents Using an Online System during Three Seasons (2019/2020–2021/2022) of Thoroughbred Flat Racing in New Zealand

**DOI:** 10.3390/ani12213028

**Published:** 2022-11-03

**Authors:** Michaela J. Gibson, Kylie A. Legg, Erica K. Gee, Chris W. Rogers

**Affiliations:** 1 School of Veterinary Sciences, Massey University, Private Bag 11-222, Palmerston North 4442, New Zealand; 2 School of Agriculture and Environment, Massey University, Private Bag 11-222, Palmerston North 4442, New Zealand

**Keywords:** thoroughbred racing, steward, stipendiary report, injury, poor performance, equine welfare

## Abstract

**Simple Summary:**

In the 2019/20 Thoroughbred racing season, the system for reporting the reasons for, and the clinical outcomes of, veterinary examinations changed from a paper-based reporting process to an online system (‘Infohorse database’). The impact of this change in reporting process was examined using the official reports from three racing seasons (2019/20 to 2021/22). The introduction of the online system did not alter the incidence of reporting events or veterinary examinations. The online system did reduce the errors in reporting events (such as misspelt horse names). The online system also improved the description of the veterinary events and the reasons for the requested veterinary examination due to consistency in the descriptors used. The structured data entry framework of the online system permitted greater ability to identify or differentiate the primary clinical presentation and the incidental or associated findings. The online system provided a more structured dataset compared to the paper-based system, making it a useful tool for the monitoring of injuries and potential risk factors within Thoroughbred racing in New Zealand. Therefore, the racing industry can meet its duty of care for racehorse and jockey welfare by making evidence-based changes to the management and structure of racing.

**Abstract:**

In the 2019/20 Thoroughbred racing season, the paper-based reporting process of stipendiary steward reports was upgraded to an online system (‘Infohorse database’) to allow for the rapid entry of precise event and injury data. The objectives of this study were to describe the incident and non-incident examinations during the 2019/20, 2020/21, and 2021/22 Thoroughbred flat racing seasons in New Zealand and describe the primary injury and reporting outcomes. The introduction of the online system was associated with fewer miscoding events with horse identification (0.1%). An improvement in the definition and prompts in reporting within the online system compared with the previous paper-based system resulted in a greater frequency of non-incident examinations being undertaken. The increased frequency of reporting the clinical outcome, ‘no observable abnormalities detected’ (NOAD), demonstrates the role of stipendiary stewards to carry out routine screening. The frequency of most clinical findings, such as musculoskeletal fractures (0.5 per 1000 starts, 95% CI = 0.3–0.6), remained similar compared to previously reported data using the paper-based system. The online system provided a more structured dataset compared with the paper-based system, making it a useful tool for the monitoring of incidents, injuries, and potential risk factors within Thoroughbred racing in New Zealand. Therefore, evidence-based changes to the management and structure of racing can be undertaken to ensure the industry meets its duty of care for racehorse and jockey welfare.

## 1. Introduction

Race-day regulation in New Zealand for Thoroughbred, harness, and greyhound racing, is controlled by an independent regulatory body, the Racing Integrity Board (RIB). On race day, stipendiary stewards monitor racing and may request the examination of a horse by the nominated race day veterinarian. When a stipendiary examination is requested, a veterinary examination is undertaken with the clinical outcome formally recorded in the published stipendiary report. Stipendiary steward reports can be requested either when an “event” has occurred such as a horse fall, collision, or stumble (incident examination), or when there has been no event, but a veterinary examination of a horse is required (non-incident examination). Non-incident examinations are often requested if a horse’s health is questioned (e.g., epistaxis or poor recovery) or a horse has raced below expectations (e.g., a heavily wagered horse that failed to place in a race).

The reporting of examinations within stipendiary steward reports for Thoroughbred flat racing in New Zealand using the historical paper-based reporting system has been published and provides baseline data on race day injuries and regulatory reporting (2015/16–2016/17 seasons) [1]. The paper-based system used a series of tick boxes and free text fields that were filled in by the official veterinarian or steward. Information on the paper reports was subsequently entered into the Racing Incident Database by a race day official at the end of the race meeting. However, the lack of constraints on the input details with the paper-based system was associated with variations in the descriptions used for clinical outcomes, requiring additional categorisation. In addition, the manual entry of the paper-based reports into the electronic system was associated with a low frequency of errors, such as incorrect race dates, misspelt horse names, and misclassification of horses (i.e., harness racing horses in the Thoroughbred racing dataset), requiring time-consuming data cleaning [1,2].

In order to improve data collection quality and ease of race day reporting, the paper-based system was replaced with an app-based online system in 2019 to record race day steward reports and veterinary examinations. The app is known as the Infohorse horse incident examination database (“the online system”). The online system was an adapted version of the Australian Racing Incident Database (ARID) introduced to racing in Australia in 2009 [3,4]. The online system was designed to provide a relatively quick method of entering race day events and injury data with greater precision. With drop-down boxes to select, the consistency of descriptors used to describe the anatomical locations and clinical outcomes of veterinary examinations can be improved and provides a clearer dataset for long-term surveillance. Therefore, greater precision in quantifying changes in the injury incidence rates with changes in race day regulation and processes can occur.

In 2019, a review of the New Zealand racing industry suggested that the number of tracks within a geographical area be consolidated to reduce the maintenance costs of tracks [5]. The initial closure of tracks resulted in seven tracks being decommissioned, of which three were in the North Island and four were in the South Island. Profits made through the selling of land could then be invested in upgrading existing tracks and the introduction of synthetic tracks. In the 2020/2021 season, New Zealand’s first synthetic track in Cambridge was opened for racing and in the 2021/22 season, the Riccarton synthetic track was opened.

Comparison of the new online system with the old paper-based system within the harness racing code identified a shift in the classification of incident examinations to non-incident examinations [2]. It was suggested that this change in reporting was a result of an improved definition of the terms incident and non-incident examination. The frequency of no observable abnormalities detected (NOAD) as a clinical finding increased in both incident and non-incident examinations, indicating that most examinations were associated with routine screening rather than an “event”. However, due to differences in racing speed, gait, and risk factors, changes in the harness racing incident reports are unlikely to reflect what has occurred in Thoroughbred racing [1].

Therefore, the objectives of this study were to describe the incident and non-incident examinations during the 2019/20, 2020/21, and 2021/22 Thoroughbred flat racing seasons in New Zealand and describe the primary injury and reporting outcomes. A secondary aim was to examine horse and race-level variables that were associated with the rate of reporting these clinical outcomes. The effect of a nine-week cessation in Thoroughbred racing during the 2019/20 racing season, due to a nationwide COVID-19 lockdown, on racing periodicity and the frequency of incident and non-incident reporting was also examined.

## 2. Materials and Methods

### 2.1. Online System—Infohorse Horse Incident Examination Database

Unlike the historical paper-based system, the online system utilised a sequence of drop-down boxes to provide prompts for the steward and/or veterinarian throughout the reporting process until all compulsory fields were entered. The online system permits entry of data directly into the Infohorse horse incidence examination database, the structure of which is based on the Australian Racing Injury Database (ARID). Additional information regarding the structure and flow of information entry within the ARID system is described by Cameron [3].

In brief, the selection of whether a report was an incident or non-incident examination determined the following sequence of reporting options. If an incident occurred, compulsory fields describing the event included: what happened (e.g., horse fell or horse was kicked), when the event happened (pre, post, or during the race), where the event occurred (on course, off course, at the barrier, at the day stalls), cause of the incident (fell, stumbled etc.), the outcome of the examination (did not complete race, scratched etc) and what was impacted (other horse, ground, etc). Non-incidents represent routine screening of a horse for reasons such as a horse performing below expectations, generally at the completion of the race and thus did not specify an event associated with the request for examination. All examinations were carried out by the designated on-duty veterinarian. If the response to the dropdown “finding” was “yes”, compulsory drop-down boxes for the body systems cardiovascular, gastrointestinal, integument, multiple, musculoskeletal, respiratory, and unknown appeared [3]. Within these body system categories, the veterinarian is then provided with prompts to ensure specific and consistent clinical findings and anatomical locations are recorded. Multiple findings could be reported for one examination. All reports as a minimum required the following information to be included: place the examination took place, the reason for veterinary examination, findings, and whether the horse was lame or not [3]. The dataset also contained a comments section where additional notes on clinical findings could be added.

### 2.2. Data Acquisition

Data for all race starts for the 2019/20, 2020/2021, and 2021/22 racing seasons were obtained as a Microsoft Excel (Microsoft Corporation, Redmond, WA, USA) spreadsheet from New Zealand Thoroughbred Racing Inc (the official registration body for Thoroughbred racing in New Zealand). These data described the racing conditions (track condition, race length, race time, number of starters, etc) and were obtained from the official race start records and subsequently cross-referenced with the stipendiary steward reports.

An Excel spreadsheet containing an extract of stipendiary steward reports from the online system was acquired from the Racing Integrity Boards (RIB). Data in this extract contained all the completed fields, including; the date, the racecourse, race number, horse name, and other detailed information relating to the non-incident/incident examination, such as the reason for the requested report and the main finding of the veterinary examination (clinical finding and anatomical location). In addition, the extract contained the free text note fields where additional notes about clinical findings were written.

The cleaned dataset, including the race level results and stipendiary steward reports for the 2015/16 and 2016/17 seasons used by Gibson, et al. [1], was retrieved to enable the comparison of clinical findings from historical data using the old paper-based system with the new online system used in the current dataset.

### 2.3. Statistical Analysis

#### 2.3.1. Data Cleaning

Stipendiary steward reports and official race start records were merged by horse name and race date to provide information about track condition (fast, good, dead, slow, heavy), horse age, and other relevant information. Track conditions were defined based on the penetrometer reading as “fast” (penetrometer reading 0.5–1.9), “good” (penetrometer reading 2.0–2.5), “dead” (penetrometer reading 2.6–3.5), “slow” (penetrometer reading 3.6–4.5), and “heavy” (penetrometer reading 4.6+) [6]. Race distance groups were defined based on race distance as “sprinter” (≤1400 m), “miler” (1401–1799 m), “middle distance” (1800–2099 m), and “stayer” (>2100 m). Race type was defined as either black type (group and listed races) or not black type (maiden, open, and rating races).

Two reports had a misspelt horse name which required correcting prior to merging with the master dataset.

Clinical outcomes generated for the 2015/16 and 2016/17 racing seasons published by Gibson, et al. [1] were used as a proxy for the old paper-based system. This method is referred to as the “notes field derived” in the current study. For the online system classification of the 2019/20, 2020/21, and 2021/22 seasons, clinical outcomes were the output of the drop-down “finding notes” menu on the online system. These data are referred to as the “drop-down system”. Clinical outcomes for both methods were categorised into 9 categories; arrhythmia/cardiovascular, cardiac failure, laceration/abrasion, lame, musculoskeletal (MS) fracture, other musculoskeletal issues, no observable abnormalities detected (NOAD), poor recovery, respiratory issues, previous injury, bleeders (epistaxis), and miscellaneous.

#### 2.3.2. Data Analysis

The distribution of median races and race starts by month between seasons was examined using a Kruskall–Wallis test. The incidence rate ratio of the occurrence of an incident examination or non-incident examination and 95% confidence intervals (95% CI) at a univariable and multivariable levels were calculated for the following variables; year (2019/20, 2020/21, and 2021/22), age category (2, 3, and 4+ years old), sex (male [colt, stallion, or gelding] and female [filly or mare]), number of starters in the race (<9 and ≥9), race distance (sprinter, miler, middle distance, and stayer), and black type race (yes or no) using Poisson regression in a generalised linear mixed model. Variables were screened in univariable models and if they reached the significance threshold (*p* < 0.2), they were included in the multivariable level. The multivariable model was built using a backwards selection procedure whereby variables that improved the model, based on a Chi-squared likelihood ratio test (*p* ≤ 0.05), were retained in the model.

Incidence rates of an event occurring were described as the number of events per 1000 starts with 95% confidence intervals [7]. Differences in the reporting rates of clinical outcomes between the 2015/16 and 2016/17 racing seasons (paper-based system) and the 2019/20, 2020/21, and 2021/22 seasons (online system) were examined. *P*-values for differences between clinical outcomes using the notes field derived and drop-down systems were calculated using Poisson regression in a generalised linear mixed model. Factors that were significant in the multivariable models for incident and non-incident examinations were included in the model with the exception of season and black-type race. To test if there was an increasing proportion of older horses racing, horses were grouped into 2 age groups: <6 years old and ≥6 years old (based on the median age). The age distribution of racing horses was determined using Simpson’s Diversity Index using these 2 groups of horses as described by Legg, et al. [8]. As the evenness of the number of racing horses in each group increases, Simpson’s Diversity Index increases.

All statistical analyses were conducted using R 3.5. 1, 2018; (R Foundation for Statistical Computing, Vienna, Austria). The R packages used were “psych” for summary data [9], “car” for the general linear models [10], and “epitools”, for incidence rates [11]. For all analyses, a level of significance was set at *p* < 0.05.

## 3. Results

Over the 2019/20-2021/22 racing seasons, there were 1903 veterinary examinations. After merging the datasets, 200 (10.5%) veterinary examinations were removed due to the horse not starting in the race (entered the racecourse and were examined by the race day veterinarian but were withdrawn from the race, n = 197), or the race/race day was abandoned (n = 3). Of the horses that were withdrawn from the race, 109 veterinary examinations (59 incident and 50 non-incident) were carried out on the course or at the barrier prior to the start of the race.

### 3.1. Thoroughbred Racing

**Figure 1:**
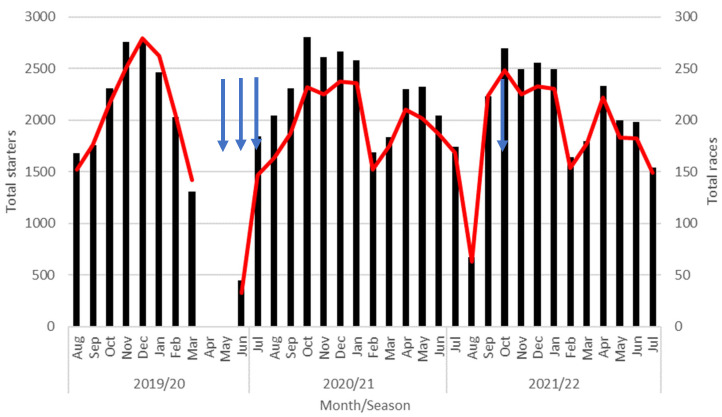
Total starters (black, left axis) by month with number of races (red, right axis) for Thoroughbred flat racing in the 2019/20–2021/22 seasons. Arrows indicate months where there was a nationwide COVID-19 lockdown.

There were 70,720 starts over the 2019/20–2021/22 racing seasons of which 19,347 (27.4%) were in the 2019/20 season, 26,934 (38.1%) were in the 2020/21 season, and 24,439 (34.6%) in the 2021/22 season. There were fewer races and race days in the COVID-19 lockdown affected season (2019/20, 1864 races and 228 race days) compared to the 2020/21 (2374 races and 277 races days) and 2021/22 seasons (2290 races and 275 race days) due to the 13-week suspension (23 March 2020 to 20 June 2020) in racing during the COVID-19 lockdown (Figure 1). In addition, there was a 19-day nationwide suspension in racing during the 2021/22 season (14 August 2021–1 September 2021) due to a second COVID-19 lockdown resulting in a decrease in total starts and races (Figure 1). In addition, there was continued regional restrictions of movement into the Auckland region until 17 January 2022 resulting in a period of 156 days when there was the ability to continue to train but no opportunity to race within the Auckland region.

Races were held on 43 racecourses with 40.8% (2666/6528 races) of races occurring in the northern region (upper North Island), 31% (2026/6528 races) of races occurring in the central region (lower North Island), and 28.1% (1836/6528 races) occurring in the southern region (South Island). Most races were undertaken on turf tracks (96.8%, 6316/6528 races) with the remaining races occurring on a synthetic track (3.2%, 212/6528 races). Of the races undertaken on turf tracks, a large proportion of races were undertaken on “dead” tracks (41.2%, 2602/6316 races), followed by “heavy” (21.4%, 1353/6316 races), “good” (17.3%, 1092/6316 races), “slow” (16.6%, 1048/6316 races), and “soft” (3.5%, 221/6316 races).

Races had a median distance of 1400 m [IQR- 1200–1600] with most races being classified as sprint races (56.6%, ≤1400 m) followed by miler (22.6%, 1401–1700 m), middle distance (8.4%, 1701–2099 m), and staying races (12.5%, ≥2100 m).

There were 7954 horses that had at least one race in at least one season. Almost half of the racing population were mares and fillies (49.5%) followed by geldings (48.1%), then stallions and colts (2.3%). Population distribution by age, sex, and season is described in Table 1.

The proportion of race starters aged <6 years old compared to ≥6 years old decreased in the 2021/22 season compared with the 2019/20 and 2020/21 seasons (Table 2, *p* < 0.001) as shown by the Simpsons diversity index (indicating a decrease in the relative proportion of older race starters).

Horses participated in a median of four races [IQR 2–6] in the 2019/20 season and five races [IQR 2–8] in the 2020/21 and 2021/22 seasons. Younger horses had fewer starts per season than older horses, with two-year-olds participating in a median of two starts [IQR 1–3], three-year-olds in a median of four starts [IQR 2–6], and horses four years and older in a median of five starts [IQR 3–8] per season. The median starter age was four years old [IQR 4–6] (*p* < 0.001).

There was a median of 11 [IQR 9–13] starters in a race over all three seasons. There were fewer starters per race (10 [IQR 8–12]) in 2019/20, than in the 2020/21 season (12 [IQR 10–13]), or the 2021/22 season (11 [IQR 9–13] starters, *p* = 0.006). There appeared to be a post-lockdown rebound in the median number of horses in a race in the months following the COVID-19 lockdown (Table 3, Supplementary Figure S1). The median number of races at a race meeting was greater in the 2019/20 season [10, IQR 8–11] compared to the 2020/21 [8, IQR 7–8] and 2021/22 [8, IQR 7–8.5] seasons (*p* < 0.001).

### 3.2. Number of Reports

During the 2019/20–2021/22 racing seasons, there were 1703 veterinary examinations (24.1 examinations per 1000 starts, 95% CI = 23.0–25.2). Most veterinary examinations were for non-incident examinations (n = 1508, 88.5%) representing routine screening of a sample of horses participating in racing. The remaining veterinary examinations were for incident examinations (n = 195, 11.5%), representing screening due to events occurring before, during, and after the race. The majority of veterinary examinations occurred post-race (n = 1611, 94.6%) with the remaining pre-race (n = 92, 5.4%).

There were more veterinary examinations in the 2020/21 (21.1 per 1000 starts, 95% CI = 19.4–22.8), and 2021/22 (25.3 per 1000 starts, 95% CI = 23.4–27.4) seasons compared with 2019/20 (26.7 per 1000 starts, 95% CI = 24.5–29.1) (*p* < 0.001). However, the incidence rate ratio of incident examinations in the 2019/20 season was greater than the 2020/21 and 2021/22 seasons (Table 4).

Within both incident and non-incident examinations, there were 32 (1.9%) clinical outcomes coded as musculoskeletal fracture (0.5 per 1000 starts, 95% CI = 0.3–0.6), 3 (0.2%) coded as cardiac failures (0.04 per 1000 starts, 95% CI = 0.00–0.13), and 136 (8.0%) coded as an epistaxis episode (1.92 per 1000 races, 95% CI = 1.63–2.28). The majority of fractures occurred in the distal limb with metacarpal (n = 10, 31.3%), metatarsal (n = 3, 9.4%), sesamoid (n = 3, 9.4%), and proximal phalanx (n = 6, 18.8%) fractures accounting for 68.8% of race day fractures. The remaining fractures were in the pelvis (n = 1, 3.1%), tibia (n = 2, 6.3%), humerus (n = 4, 12.5%), and radius (n = 2, 6.3%).

#### 3.2.1. Incident Examinations

An incident report was requested 2.7 per 1000 races (95% CI = 2.4–3.2). Most incident examinations were associated with a turf track (95.9%, 187/195 incident examinations) with the remaining examinations for races on synthetic tracks (4.1%, 8/195 incident examinations). Of the incident examinations on turf tracks, almost half were on “dead” tracks (47.6%, 89/187), followed by “good” (21.9%, 41/187), “heavy” (16.0%, 30/187), “slow” (10.7%, 20/187), and “soft” (3.7%, 7/187). There was an association between racing season and the frequency of incident examinations (Supplementary Table S1, *p* > 0.05). In addition, the number of starters and track condition met the threshold (*p* < 0.2) to be included in the multivariable model (Table 4). In the multivariable model, seasons continued to have an association with the frequency of incident reporting, such that the 2019/2020 season had greater incidence of an incident examination being carried out. In addition, track condition had an association, such that an incident examination was less likely to be requested on a “slow” track.

Reasons for an incident examination being requested included “pulled up” (n = 31, 15.9%), “horse fell” (n = 25, 12.8%), “fractious behaviour” (n = 24, 12.3%), “galloped on” (n = 20, 10.3%), “kicked” (n = 11, 5.6%), “rider fell” (n = 6, 3.1%), and “loose horse” (n = 4, 2.1%). Just over a third of the incident examinations did not have a coding category describing why the incident report was requested (other, n = 74, 37.9%). Examination of the free text comments section revealed that many of these uncategorised reports were often for events that did not fit the given categories, such as a horse hitting its head in the starting gates.

Half of the incident examinations had NOAD listed as the clinical outcome (n = 98, 50.3%, Table 5). The most common clinical finding was laceration/abrasion (n = 49, 25.1%) followed by musculoskeletal fracture (n = 17, 8.7%) and other musculoskeletal issues (n = 15, 7.7%).

There were 17 musculoskeletal fractures and one cardiac failure clinical outcomes within incident examinations using the drop-down system. In addition, there were 10 (probable = 2, confirmed = 8) musculoskeletal fractures described in the free text notes/comments section that were not reported in the drop-down field (no finding, n = 6), or classified as musculoskeletal other (n = 4). There were two musculoskeletal other clinical outcomes for joint dislocations which resulted in the horses being euthanised. One incident examination had a clinical outcome of “cardiovascular” but stated that the horse died in the “event outcome”. Another incident report stated that there was no clinical finding, but in the manual entry notes the horse had died from a suspected internal haemorrhage.

The use of the drop-down system was associated with a decrease in the frequency of clinical outcomes coded as cardiovascular, whilst the frequency of clinical outcomes coded as respiratory increased compared with the previously reported 2015/16–2016/17 season note field-derived system.

#### 3.2.2. Non-Incident Examinations

A non-incident examination occurred 21.3 (95% CI = 20.3–22.4) times per 1000 races. The main reason for a non-incident examination to be requested was for poor performance (77.2%, 1165/1508) and performed below expectations (6.8%, 102/1508) (combined accounted for 84.0%, 1267/1508). Other reasons for a non-incident examination were injury (5.8%, 87/1508), routine inspection (1.6%, 24/1508), incident (1.1%, 17/1508), condition disclosed (0.5%, 8/1508), and not disclosed (0.5%, 8/1508). The remaining non-incident examinations were classified as “other” (6.4% 97/1508). Stewards were responsible for requesting the majority of non-incident examinations (87.7%, 1323/1508), followed by trainers (8.4%, 126/1508), veterinarians (2.5%, 38/1508), jockeys (0.9%,13/ 1508), and starting personnel (0.5%, 8/1508). Horses in a race with nine or more starters were less likely to have a non-incident examination than horses in a race with less than nine starters (Supplementary Table S2). There were 93 horses over the three seasons that had two non-incident examinations for poor performance, 17 horses with three records, and three horses with four records.

Within the reasons for non-incident examination coded as poor performance or performed below expectations, the most common clinical outcome was NOAD (969/1267, 76.5%). The majority of non-incident examinations had NOAD listed as the clinical outcome (Table 5). The most common clinical findings for a non-incident examination were epistaxis (8.42%) followed by laceration/abrasion (6.76%) and musculoskeletal other (5.24%).

There were three non-incident examinations with the clinical outcome of musculoskeletal fracture and none for cardiac failure. There were an additional two non-incident examinations that suggested a musculoskeletal fracture in the manual comments section. Of these examinations, one was classified in the drop-down clinical outcomes as “other musculoskeletal issue” and the other had no clinical finding in the drop-down section.

The proportion of clinical outcomes for a non-incident event with NOAD or epistaxis listed as the clinical outcome increased from that previously reported in the notes field-derived system (Table 5). The frequency of lacerations/abrasions, musculoskeletal fractures, poor recovery, and miscellaneous clinical outcomes was less than the notes field-derived system.

At the univariable level, there was an association between season, age category, number of starters, track condition, race distance, and race type and the frequency of non-incident examinations; therefore, they were included in the multivariable model (Supplementary Table S2). All variables identified at the univariable level remained in the final multivariable model (Table 6).

## 4. Discussion

The racing season in which there was the introduction of the Infohorse incident examination online system coincided with the racing season affected by a nationwide lockdown to control the spread of COVID-19 in New Zealand. This provided the opportunity to examine the impact the online system had on the recording process and examine if an enforced break in racing and training was associated with a shift in the racehorse injury profile reported once racing recommenced. The introduction of the online system was associated with a greater consistency in the descriptors of injuries and race day events. As identified with a similar dataset for harness racing horses [12], one of the major outcomes of this consistency was the correct classification of injuries associated with racing events (i.e., incident examinations). In contrast to our hypothesis, despite a subtle change in the pattern of racing after the COVID-19 lockdowns and associated break in racing and training, there were no changes in the type or incidence of injuries reported.

Changes in racing venues and the introduction of the synthetic track did not affect the distribution of races within regions in New Zealand compared with previously reported data [1]. This result is similar to harness racing, where the reduction in racetracks did not affect the racing profile, as clubs affected by racecourse closures continued to hold race meetings but at adjacent racecourses [12].

Overall, the racing population had a similar number of horses and age profile as previously reported for the 2015/16–2016/17 seasons. The increase in age profile follows a similar trend to what has been previously reported by Legg et al. [8] indicating a growing population of older horses. There appeared to be a post-COVID-19 lockdown rebound in racing starts. In comparison to the reference years for the period June and July, there was an increase in the number of horses per race and races per race meeting. Some industry observers attributed this, and the sustained increase in interest in racing during the following season, to be due to associated international travel restrictions (no overseas holidays) and therefore effectively more discretionary income available for investment in racing.

Unlike harness racing, the training of Thoroughbreds in New Zealand is primarily undertaken at centralised training locations. Therefore, during the first COVID-19 lockdown (2019/20 season), the majority of Thoroughbred horses were unable to remain in training and were “turned-out” for a spell. When racing could resume, there was a lag in the time it took for horses to return to racing, associated with the time required to regain race fitness. Therefore, Thoroughbred racing had a longer non-racing period of 89 days, than the 67-day break in racing observed for harness racing [12].

The introduction of the online system was associated with an increase in the frequency of non-incident examinations reported. The incidence rate of non-incident examinations for Thoroughbred flat racing was similar to harness racing for the same seasons (unpublished data), reflecting the consistency in the regulatory process across the two racing codes and seasons [1,2]. However, the decrease in the incidence of reports coded as incident examinations within harness racing with the introduction of the online system was not as acute in the current Thoroughbred dataset (unpublished data). The decrease in the frequency of incident examinations is suggested to be due to greater clarity of the definition of an incident and non-incident examination.

There was no increase, or change in the type, of race day injuries reported immediately after the COVID-19 lockdown-enforced break in training and racing. This was surprising given the association within the literature of returning from a spell and injury and fracture risk [13,14,15]. It may be that the incidence rates reported in the published data are biased upwards due to horses with predisposing factors for fracture being more likely to be spelled. In contrast, the enforced spell in our population was due to variables extrinsic to the trainer. Many racehorses in New Zealand are also spelled in large paddocks and thus given the opportunity to maintain some degree of fitness and bone strength via bursts of osteoinductive play [16,17]. The enforced break in training was effectively the 67 days associated with the lockdown followed by 22 days of training before the first race start, which was less than the 161 days break between races usually associated with racehorses in New Zealand [8]. The rapid resumption of horse numbers racing post-lockdown also implies that many of the horses may have been receiving some work during the lockdown as they only had 21 days to regain race fitness.

A greater incidence rate ratio of an incident examination being requested in the 2019/20 season compared with the 2020/21 and 2021/22 seasons may be due to the rapid return to racing of large cohorts of horses after the COVID-19 lockdown. In addition, the greater incidence rate of incident examinations in the 2020/21 season compared with the 2021/2022 season may be a carryover effect of the resumption of racing at the end of the 2019/20 season. At the resumption of racing, it is possible that some horse’s behaviour, due to being “fresh” or “out of practice” resulted in a greater likelihood of an incident occurring. In addition, the post-lockdown rebound resulted in a greater number of starters in a race and a reduction in races with few starters. An increase in the number of horses in a race is known to increase the likelihood of an event such as a horse colliding with another horse [15,18].

The decrease in the incidence of lacerations and abrasions in non-incident reports compared with the previously reported 2015/16–2016/17 seasons [1], may reflect a change in the pattern of reporting due to structured data entry in the online system. With the previous paper-based reporting system, the ability to determine the significance of a laceration or abrasion was often limited by what was written within the notes section, so for coding purposes, any mention of a laceration or abrasion was previously considered a significant finding. Within the online system for any clinical outcome to be classified as a significant finding, the drop-down box called “finding” must be answered with “yes” to enable the drop-down boxes describing the clinical outcome to become available. The ability for the veterinarian to classify the clinical outcome increased the incidence of epistaxis being reported. Within the old paper-based system, if epistaxis was not specifically stated within the notes fields, then the appropriate clinical coding may have resulted in the incidence being underreported. The incidence rate of epistaxis reported from data collected with the online system now is in closer agreement with figures reported from Australia [19] and the United Kingdom [20].

The track conditions associated with a lower incidence rate of both incident and non-incident examinations were heavy and slow tracks. This result is in agreement with previously reported data [1,15]. Races held on softer tracks tend to have a lower risk of injury, likely due to the slower racing conditions [20]. During wet months (i.e., winter), races tend to be held on courses that are free draining to provide consistent racing conditions [18]. In addition, the stipendiary stewards have the ability to postpone or cancel a race meeting due to adverse track conditions. As a result, injuries due to poor track conditions can be limited [2]. Only a small proportion of races (3.2% of all races) were carried out on synthetic tracks as the two tracks were opened for racing in the 2020/21 and 2021/22 seasons. A higher IR of non-incident examinations on synthetic tracks may be due to horses adapting to racing on the new surface. Horses that have not trained on a synthetic track prior to racing may take time to adapt to the new surface. Due to the recent introduction of the synthetic tracks, the ability to compare incident reporting between grass and synthetic tracks was limited and requires additional seasons of data to compare the risks associated with track surfaces.

The effect of age and race type on non-incident reporting reflects the effect of horse experience on performance. Younger horses were more likely to have a non-incident examination requested, likely due to inexperience affecting performance [21]. Horses are retained in racing if they perform well and are not affected by health conditions. Therefore, older horses retained in racing are less likely to have a non-incident examination requested for poor performance or a health concern (i.e., a healthy horse effect) [14]. Similarly, horses racing in non-black type races tend to be less experienced or of a lower standard than horses in black-type races, so are more likely to perform poorly and have a non-incident examination requested than the higher-ranked horses in black-type races. Horses in black-type races also tend to be ridden by more experienced jockeys, maximising horse performance and reducing jockey error contributing to horse performance [21].

The decrease in miscellaneous findings in the non-incident examinations indicates that the drop-down system provides a greater opportunity to classify clinical outcomes. The drop-down system resulted in the classification of over 99% of the clinical outcomes for incident and non-incident examinations. However, the misclassification of some musculoskeletal fractures and cardiac failures suggests that the reporting system could be refined. In addition the interchangeable use of “poor performance” and “performed below expectations” means there is space for additional definitions to be provided [2].

A number of limitations should be noted. The introduction of the online system and the advent of the COVID-19 lockdown and enforced break in training and racing occurred in the first year of the observation period reported in this study. The 2020/21 and 2021/22 racing seasons thus became the control seasons for examining the effect of the COVID-19 interruption on injury reporting. It is possible that these two seasons in themselves may not be “typical years” as there may be some carryover from the COVID-19-associated interruption and associated racing rebound. However, the pattern of racing described for these two seasons was similar to the historical data reported by Legg et al. [8] and in the years immediately prior to the COVID-19 lockdown [1].

As a retrospective study, the variables able to be examined were constrained by the data regularly recorded within the racing industry. It is widely recognized that injuries reported on race day represent only race day-associated injuries and not training-related injuries, which represent the greatest proportion of injuries to racehorses. The collection of training data would be a useful addition to understanding the interaction of training load on race day injury. However, this was not possible with a retrospective study, and training data is not regularly reported or summarized in New Zealand in a form suitable for epidemiological studies. Retrospective collection of training data from trainers is also fraught with recall bias and lack of precision in the reporting of distances covered and associated speeds.

## 5. Conclusions

The introduction of the online system provided greater consistency in the way incidents and injuries were reported, but did not appear to signal a change in the frequency of major injuries. However, minor injuries, often secondary to the injury of interest, such as lacerations, may have been overemphasised or reported via the paper-based system as there was no opportunity to clearly identify the primary injury and reporting of secondary findings, such as lacerations or cuts, within the free text descriptors. The online system utilised fields and reporting structure similar to that routinely used in Australia with the ARID system and going forward, this may permit use of larger Australasian racing and injury data to identify risk factors and efficacy of changes in regulatory processes that are aimed at improving the safety and welfare for both racehorses and jockeys. The inclusion, or ability of the trainer’s treating veterinarian to enter data, such as findings from requested follow up veterinary examinations or monitoring, would greatly improve our understanding of the implications of some of these race day events and provide greater transparency in the management of racehorses.

## Figures and Tables

**Table 1:** Population distribution of horses participating in the 2019/2020, 2020/2021, and 2021/22 Thoroughbred flat racing seasons in New Zealand including number of horses per season, starters per season, and sex of horses by age category.

	Horses per Season			Starters per Season			Sex Descriptor (Number of Horses)				
Age (Years)	2019/20	2020/21	2021/22	2019/20	2020/21	2021/22	Colts	Fillies	Geldings	Entires/ Stallions	Mares
2	230	314	306	464	741	696	121	494	249		
3	966	1224	1148	3664	5331	5066	139	1852	1403		
4	1132	1151	1247	5192	7172	7272			1632	29	1883
5	912	869	754	4773	6125	5064			1226	9	1301
6	452	590	489	2496	3943	3242			825	6	701
7	282	284	270	1558	1889	1845			538	2	296
8	136	176	101	752	1128	614			310	4	99
9	56	80	76	299	409	480			176	3	33
10	35	29	28	128	138	115			81		11
11	9	12	10	19	52	42			31		
12	1	3	2	1	5	3			6		
13	1	1	0	1	1	0			2		
Total	4212	4733	4431	19,347	26,934	24,439	260	2346	6479	53	4324

**Table 2:** Number of race starts, percentage of total starts, and Simpson’s index for Thoroughbred flat racing horses <6-years-old and ≥6-years-old for the 2019/20, 2020/21, and 2021/22 seasons.

Season	Starters < 6-Year-Old	Starters ≥ 6-Year-Olds	Simpsons Index
2019/2020	14,093 (72.8%)	5254 (27.2%)	0.40
2020/2021	19,369 (71.9%)	7565 (28.1%)	0.40
2021/2022	18,098 (74.1%)	6341 (25.9%)	0.38

**Table 3:** Descriptive race-level data for the months of June and July representing the post-COVID-19 lockdown rebound in racing (2019/20 season) and the 2020/21 and 2021/22 seasons as reference values.

	2019/20		2020/21		2021/22	
	June	July	June	July	June	July
Horses racing	444	1388	1402	1159	1396	1089
Number of starts	445	1841	2042	1741	1984	1543
Number of races	33	147	186	168	182	149
Median of starters/race *	14 [12–15]	13 [11–14]	11 [10–12.8]	10 [8–12]	11 [9–13]	10 [9–12]
Median races/meeting *	11 [11–11]	9 [8–11]	8 [7.75–8.25]	8 [7–8]	8 [7–9]	8 [7–8]

* Data presented as median and interquartile values.

**Table 4:** Multivariable incidence rate ratios (IRR) and 95% confidence of an incident (horse fall, collision, or stumble) examination occurring with the effects of season and track surface.

Multivariable	Incidents	Starts	IRR	*p*-Value	Wald *p*-Value
Season					
2019/20	74 (37.9%)	19,347	(Referent)		0.004
2020/21	66 (33.8%)	26,934	0.7 [0.5–0.9]	0.015	
2021/22	55 (28.2%)	24,439	0.6 [0.4–0.8]	0.002	
Surface					
Dead	89 (45.6%)	28,562	(Referent)		0.051
Good	41 (21.0%)	11,670	1.1 [0.7–1.6]	0.677	
Heavy	30 (15.4%)	14,177	0.7 [0.4–1.0]	0.069	
Slow	20 (10.3%)	11,538	0.6 [0.3–0.9]	0.024	
Soft	7 (3.6%)	2406	1.2 [0.5–2.6]	0.637	
Synthetic	8 (4.1%)	2172	1.5 [0.6–2.9]	0.313	

**Table 5:** Incidence of clinical findings (incidence rate; IR) in incident (horse fall, collision, or stumble) and non-incident (veterinary examination of a horse is required but no extenuating circumstances) veterinary examinations over four complete Thoroughbred flat racing seasons using the notes field-derived system for the 2015/16–2016/17 seasons and the drop-down system for the 2019/20–2020/21 seasons. Data are reported as incidence per 1000 starts (with 95% confidence intervals) and the percentage of clinical findings (%) within the respective report type (incident or non-incident).

	Incident Examinations				Non-Incident Examinations					
	2015/16–2016/17 Data		2019/20–2021/22 Data		2015/16–2016/17 Data			2019/20–2021/22 Data		
Description	IR	%	IR	%	*p*-Value	IR	%	IR	%	*p*-Value
Total reports	3.27 [2.82–3.79]		2.76 [2.40–3.17]		0.054	15.38 [14.38–16.44]		21.32 [20.28–22.41]		<0.001
Cardiovascular	0.16 [0.08–0.33]	5.0	0.02 [0.00–0.11]	1.0	0.023	0.74 [0.55–1.02]	4.9	0.89 [0.69–1.14]	4.2	0.362
Cardiac failure	0.05 [0.01–0.02]	1.7	0.01 [0.00–0.01]	0.5	0.219	0.02 [0.00–0.11]	0.1	0.00	0.0	0.991
Laceration/abrasion	0.69 [0.50–0.96]	21.2	0.69 [0.52–0.92]	25.1	0.905	2.23 [1.87–2.66]	14.5	1.44 [1.19–1.75]	6.8	<0.001
Lame	0.18 [0.01-0.34]	5.6	NA *	0.0		1.15 [0.90–1.48]	7.5	NA *	0.0	
MS fracture	0.18 [0.01–0.34]	5.6	0.24 [0.15–0.39]	8.7	0.437	0.38 [0.25–0.59]	2.5	0.04 [0.00–0.13]	0.2	<0.001
Other MS issues	0.29 [0.18–0.48]	8.9	0.21 [0.13–0.35]	7.7	0.300	0.97 [0.74–1.27]	6.3	1.12 [0.90–1.39]	5.2	0.394
NOAD	1.26 [1.00–1.60]	38.6	1.39 [1.14–1.69]	50.3	0.824	7.19 [6.51–7.93]	46.7	15.0 [14.15–15.94]	70.4	<0.001
Poor recovery	0.05 [0.01–0.17]	1.7	0.01 [0.00–0.09]	0.5	0.286	0.79 [0.58–1.06]	5.1	0.42 [0.29–0.61]	2.0	0.030
Respiratory issues	0.00	2.2	0.07 [0.02–0.20]	0.0	0.993	0.62 [0.44–0.87]	4.0	0.45 [0.32–0.64]	2.1	0.878
Previous injury	0.04 [0.00–0.14]	1.1	NA *	0.0		0.05 [0.01–0.17]	0.4	NA *	0.0	
Epistaxis	0.20 [0.11–0.37]	6.2	0.13 [0.06–0.25]	4.6	0.179	0.80 [0.60–1.08]	5.2	1.80 [1.51–2.14]	8.4	<0.001
Miscellaneous	0.07 [0.02–0.20]	2.2	0.01 [0.00–0.09]	0.51	0.146	0.42 [0.28–0.63]	2.73	0.14 [0.07–0.26]	0.66	0.002

* NA- not applicable as clinical outcome was not present on the drop-down system.

**Table 6:** Multivariable incidence rate ratios (IRR) and 95% confidence of a non-incident (veterinary examination of a horse is required but no extenuating circumstances) examination occurring with the effects of season, age category, number of starters, track surface, race distance, and race type.

Multivariable	Non-Incidents	Starts	IRR	*p*-Value	Wald *p*-Value
Season					
2019/20	443 (29.4%)	19,347	(Referent)		0.007
2020/21	501 (33.2%)	26,934	0.8 [0.7–0.9]	0.004	
2021/22	564 (37.4%)	24,439	1.0 [0.8–1.1]	0.582	
Age category (years)					
2	61 (4.0%)	1901	(Referent)		<0.001
3	345 (22.9%)	14,061	0.8 [0.6–1.0]	0.049	
4+	1102 (73.1%)	54,758	0.6 [0.5–0.8]	<0.001	
Number of starters					
Less than 9	265 (17.6%)	9696	(Referent)		<0.001
9 or more	1243 (82.4%)	61,024	0.8 [0.7–0.9]	<0.001	
Surface					
Dead	649 (43.0%)	28,651	(Referent)		<0.001
Good	265 (17.6%)	11,711	1.0 [0.8–1.1]	0.537	
Heavy	261 (17.3%)	14,207	0.8 [0.7–0.9]	0.003	
Slow	196 (13.0%)	11,558	0.8 [0.7–0.9]	0.001	
Soft	70 (4.6%)	2413	1.2 [0.9–1.5]	0.162	
Synthetic	67 (4.4%)	2180	1.3 [1.0–1.7]	0.026	
Distance					
Sprinter	762 (50.5%)	38,839	0.9 [0.7–1.1]	0.164	<0.001
Miler	362 (24.0%)	16,774	1.0 [0.8–1.3]	0.767	
Middle distance	259 (17.2%)	6094	(Referent)		
Stayer	125 (8.3%)	9013	1.4 [1.2–1.8]	0.001	
Black type race					
Yes	2 (0.1%)	416	0.2 [0.03–0.6]		
No	1506 (99.9%)	70,304	Referent		0.022

## Data Availability

Data available from corresponding author upon request.

## Supplementary Materials

The following supporting information can be downloaded at: https://www.mdpi.com/article/10.3390/ani12213028/s1, Figure S1: Median number of starters (black bar) and races (red line) by season and month for Thoroughbred flat racing in the 2019/20-2021/22 seasons. Arrows indicate months where there was a nationwide COVID-19 lockdown. Table S1: Univariable incidence rate ratios (IRR) and 95% confidence of an incident (horse fall, collision, or stumble) examination occurring with the effects of season, age category, sex, number of starters, track surface, race distance and race type. Table S2: Univariable incidence rate ratios (IRR) and 95% confidence of a non-incident (veterinary examination of a horse is required but no extenuating circumstances) examination occurring with the effects of season, age category, sex, number of starters, track surface, race distance and race type.

## References

[1] Gibson M.J., Bolwell C.F., Gee E.K., Legg K.A., Rogers C.W. (2022). Race-Level Reporting of Incidents during Two Seasons (2015/16 to 2016/17) of Thoroughbred Flat Racing in New Zealand. Animals.

[2] Gibson M.J., Roca Fraga F.J., Bolwell C.F., Gee E.K., Rogers C.W. (2022). Race-Level Reporting of Incidents during Two Seasons (2015/16 to 2016/17) of Harness Racing in New Zealand. Animals.

[3] Cameron A. (2005). A Web-Based Racing Injury Reporting System for Human and Equine Injuries.

[4] Collier D.J. (1999). Gastric ulceration: Response to an unnatural environment. Equine Vet. J. Suppl..

[5] Messara J. (2018). Review of the New Zealand Racing Industry.

[6] Murphy J., Field T., Thomas V. (1996). Racetrack traction assessment by penetrometer part ii. application of the model. J. Turfgrass Manag..

[7] Parkin T.D. (2008). Epidemiology of racetrack injuries in racehorses. Vet. Clin. N. Am. Equine Pract..

[8] Legg K.A., Gee E.K., Cochrane D.J., Rogers C.W. (2021). Preliminary Examination of the Biological and Industry Constraints on the Structure and Pattern of Thoroughbred Racing in New Zealand over Thirteen Seasons: 2005/06–2017/18. Animals.

[9] Revelle W. (2018). Psych: Procedures for Psychological, Psychometric, and Personality Research.

[10] Fox J., Weisberg S. (2018). An R Companion to Applied Regression.

[11] Aragon T., Fay M., Wollschlaeger D., Omidpanah A. (2012). EpiTools: R Package for Epidemiologic Data and Graphics.

[12] Gibson M.J., Legg K.A., Gee E.K., Rogers C.W. Race-level reporting of incidents using the new online system during two seasons (2019/2020–2020/2021) of Harness Racing in New Zealand. J. Equine Vet. Sci..

[13] Martig S., Chen W., Lee P., Whitton R. (2014). Bone fatigue and its implications for injuries in racehorses. Equine Vet. J..

[14] Bolwell C., Rogers C., Gee E., McIlwraith W. (2017). Epidemiology of musculoskeletal injury during racing on New Zealand racetracks 2005–2011. Animals.

[15] Hitchens P., Morrice-West A., Stevenson M., Whitton R. (2019). Meta-analysis of risk factors for racehorse catastrophic musculoskeletal injury in flat racing. Vet. J..

[16] Rogers C.W., Dittmer K.E. (2019). Does Juvenile Play Programme the Equine Musculoskeletal System?. Animals.

[17] Firth E.C., Rogers C.W., van Weeren P.R., Barneveld A., McIlwraith C.W., Kawcak C.E., Goodship A.E., Smith R.K. (2012). The effect of previous conditioning exercise on diaphyseal and metaphyseal bone to imposition and withdrawal of training in young Thoroughbred horses. Vet. J..

[18] Tanner J., Rogers C., Bolwell C., Cogger N., Gee E., Mcllwraith W. (2016). Analysis of failure to finish a race in a cohort of Thoroughbred racehorses in New Zealand. Animals.

[19] Langford J., Thomson P., Knight P. (2013). Epistaxis in racehorses: Risk factors and effects on career. Aust. Vet. J..

[20] Rosanowski S., Chang Y., Stirk A., Verheyen K. (2017). Descriptive epidemiology of veterinary events in flat racing Thoroughbreds in Great Britain (2000 to 2013). Equine Vet. J..

[21] Legg K.A., Cochrane D.J., Bolwell C.F., Gee E.K., Rogers C.W. (2020). Incidence and risk factors for race-day jockey falls over fourteen years. J. Sci. Med. Sport.

