# Supplementary material for: Race-Level Reporting of Incidents Using an Online System during Three Seasons (2019/2020–2021/2022) of Thoroughbred Flat Racing in New Zealand
Source: Animals (Basel). 2022 Nov 3;12(21):3028. doi: 10.3390/ani12213028 (PMC9658589; doi:10.3390/ani12213028)
Supplement: Supplementary file 1 [file animals-12-03028-s001.zip › animals-1922970-supplementary.pdf]

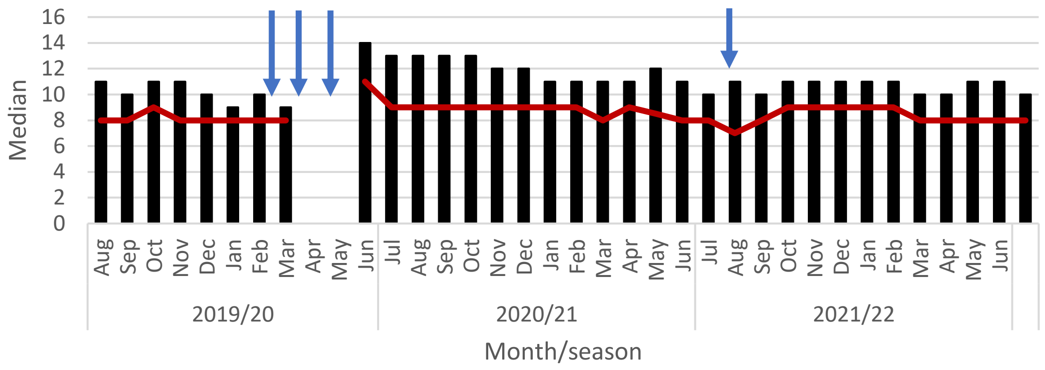

Supplementary Figure S1. Median number of starters (black bar) and races (red line) by season and month for Thoroughbred flat racing in the 2019/20-2021/22 seasons. Arrows indicate months where there was a nationwide Covid 19 lockdown.

Supplementary Table S1. Univariable incidence rate ratios (IRR) and 95% confidence of an incident (horse fall, collision, or stumble) examination occurring with the effects of season, age category, sex, number of starters, track surface, race distance and race type.

**Univariable**	**IRR**	**p-value**	**Wald p-value**
Season			
2019/20	(Referent)		0.004
2020/21	0.6 [0.5-0.9]	0.008	
2021/22	0.6 [0.4-0.8]	0.003	
Age category (years)			
2	(Referent)		0.746
3	1.4 [0.6-4.7]	0.503	
4+	1.3 [0.5-4.2]	0.612	
Sex			
Male (colt, gelding stallion)	0.8 [0.6-1.1]		
Female (filly or mare)	(Referent)		0.242
Number of starters			
Less than 9	(Referent)		0.132
9 or more	0.8 [0.5-1.1]		
Surface			
Dead	(Referent)		0.060
Good	1.1 [0.8-1.6]	0.526	
Heavy	0.7 [0.4-1.0]	0.068	
Slow	0.6 [0.3-0.9]	0.018	
Soft	0.9 [0.4-1.9]	0.862	
Synthetic	1.2 [0.5-2.3]	0.652	
Distance			
Sprinter	1.2 [0.7-2.3]	0.480	
Miler	1.3 [0.7-2.4]	0.384	0.290
Middle distance	(Referent)		
Stayer	1.7 [0.9-3.4]	0.099	
Black type race			
Yes	0.9 [0.05-3.9]		
No	Referent		0.891

Supplementary Table S2. Univariable incidence rate ratios (IRR) and 95% confidence of a non-incident (veterinary examination of a horse is required but no extenuating circumstances) examination occurring with the effects of season, age category, sex, number of starters, track surface, race distance and race type.

Univariable	IRR	p-value	Wald p-value
Season			
2019/20	(Referent)		<0.001
2020/21	0.8 [0.7-0.9]	0.001	
2021/22	1.0 [0.9-1.1]	0.902	
Age category (years)			
2	(Referent)		<0.001
3	0.8 [0.6-1.0]	0.053	
4+	0.6 [0.5-0.8]	<0.001	
Sex			
Male (colt, gelding stallion)	1.0 [0.9-1.1]		
Female (filly or mare)	(Referent)		0.705
Number of starters			
Less than 9	(Referent)		<0.001
9 or more	0.7 [0.7-0.9]		
Surface			
Dead	(Referent)		<0.001
Good	1.0 [0.9-1.2]	0.988	
Heavy	0.8 [0.7-0.9]	0.004	
Slow	0.7 [0.6-0.9]	<0.001	
Soft	1.3 [1.0-1.6]	0.049	
Synthetic	1.4 [1.0-1.7]	0.017	
Distance			
Sprinter	1.0 [0.8-1.2]	0.645	<0.001
Miler	1.1 [0.9-1.3]	0.624	
Middle distance	(Referent)		
Stayer	1.4 [1.1-1.7]	0.002	
Race type			
Yes	0.2 [0.04-0.7]		
No	Referent		0.035
